# High-dose intravenous glucocorticoid induces hyperamylasemia: a case series

**DOI:** 10.1186/s13256-022-03588-0

**Published:** 2022-10-12

**Authors:** Di Yang, Ming-hui Li

**Affiliations:** 1 Shaoxing University School of Medicine, Shaoxing, China; 2 The First Affiliated Hospital of Shaoxing University (Shaoxing People’s Hospital), Shaoxing, China

**Keywords:** High-dose glucocorticoid, Intravenous application, Hyperamylasemia, Case report

## Abstract

**Background:**

Glucocorticoids have many side effects, and high-dose intravenous application may cause rare adverse reactions such as hyperamylasemia. The aim of this study is to explore the clinical characteristics, treatment, and prognosis of hyperamylasemia induced by high-dose intravenous glucocorticoids.

**Case presentation:**

Four Asian female patients, aged between 26 and 71 years, were diagnosed with hyperamylasemia after intravenous administration of high-dose glucocorticoid. Amylase levels were elevated to varying degrees in all patients, but the peaks were below three times the upper limit of normal, and imaging showed no significant pancreatic abnormalities. Two patients developed abdominal pain, which was resolved by inhibition of pancreatic secretion, while the other patients were asymptomatic. Two patients were discharged after a significant decrease in amylase levels, while the other two were discharged after improvement of the primary disease.

**Conclusion:**

High-dose intravenous glucocorticoid can cause hyperamylasemia, which should be given enough attention by clinicians. Etiological differentiation of hyperamylasemia should be emphasized in clinical practice, especially when the diagnosis of acute pancreatitis is not clear.

## Background

Glucocorticoids (GCs) exert antiinflammatory and immunosuppressive effects by interfering with genome transcription and through transactivation of inflammatory cytokines [1]. In addition, GCs mediate physiological activities such as activation of gluconeogenesis, insulin resistance, skin atrophy, and inhibition of bone formation, resulting in corresponding complications and adverse reactions [2]. Methylprednisolone (mPSL) is a synthetic GC and is currently the first-line drug for inflammatory and autoimmune diseases. Common mPSL adverse reactions include Cushing’s syndrome, induction and exacerbation of infections, gastrointestinal reactions, osteoporosis, and obesity [3]. High-dose intravenous administration of GCs may induce other rare adverse reactions.

This study reviewed the clinical features and treatment of four patients with hyperamylasemia who received high-dose intravenous mPSL in our hospital from 2019 to 2021. The findings of this study provide information to improve understanding of clinicians on the application of high-dose GCs. The reporting of this study conforms to CARE guidelines [4].

## Case presentation

### Patient information and clinical data

#### General information

According to the China 2020 expert consensus on emergency application of corticosteroid [4], the definition of high-dose mPSL is 1–4 mg/(kg days), which is defined as >1.5 mg/(kg days) in this paper. Hyperamylasemia refers to elevated serum amylase levels above the normal upper limit (hospital normal range 40–132 U/L) [5]. Four patients, all Asian female, aged between 26 and 71 years old, with average age of 49.25 ± 18.38 were included in this study. To protect the privacy of the patients, we hid their real names, visiting department, hospitalization number, bed number, and other related information. Cases 1 and 2 were previously healthy, while cases 3 and 4 were accompanied by hypertension and renal insufficiency. There was no obvious abnormality in their family or psychosocial history, including related genetic information. Investigations on admission showed no history of pancreatic/salivary gland-related diseases or trauma, neoplasms, genital tract diseases, endoscopic retrograde cholangiopancreatography (ERCP) operation history, or abdominal surgery. In addition, patients were not diagnosed with hyperlipidemia, alcoholism, common drug factors (such as amino salicylic acid, thiopurine, and asparaginase), or megaamylasemia that can cause hyperamylasemia [6]. Basic information and clinical data of the four patients are presented in Table 1.

**Table 1:** Clinical data

Case	Age/sex	Primary disease	Main symptoms	mPSL dose	AMY peak (U/L)	New symptoms	Other therapeutic drugs	Outcome
1	49/F	ADEM	Fever, headache, jet vomiting	500 mg/day for 7 days + 250 mg/day for 5 days + 120 mg/day for 5 days	336.9	Abdominal pain	Ceftriaxone sodium, acyclovir	Better
2	26/F	ADEM	Fever, headache, dysuria	80 mg q8h 2 days + 120 mg/day for 2 days + 200 mg/day for 2 days + 120 mg/day for 2 days	340.4	Abdominal distension, abdominal pain	Ceftriaxone sodium, acyclovir, piperacillin tazobactam	Better
3	51/F	SLE	Tooth loss, abdominal pain, dry mouth, knee joint pain	500 mg/day for 3 days	193.1	None	Cefotiam, cyclophosphamide	Discharged
4	71/F	Uremia, nephrotic syndrome	Gross hematuria, edema	250 mg/day for 3 days + 250 mg/day 3 days	257.9	None	Piperacillin tazobactam, cyclophosphamide	Discharged

*ADEM* Acute disseminated cerebrospinal meningitis,* SLE* systemic lupus erythematosus,* F* female

#### Clinical manifestations

Case 1 showed persistent upper abdominal pain and right abdominal tenderness, with no episodes of vomiting, diarrhea, or rebound pain following high-dose intravenous administration of mPSL. Case 2 had abdominal distension, persistent epigastric pain, with no other concomitant symptoms, either. Meanwhile, cases 3 and 4 did not show any significant symptoms. The positive physical examination results of the four cases were all related to the primary disease, and the abdominal physical examination showed no apparent abnormality.

#### Clinical findings

**Fig. 1:**
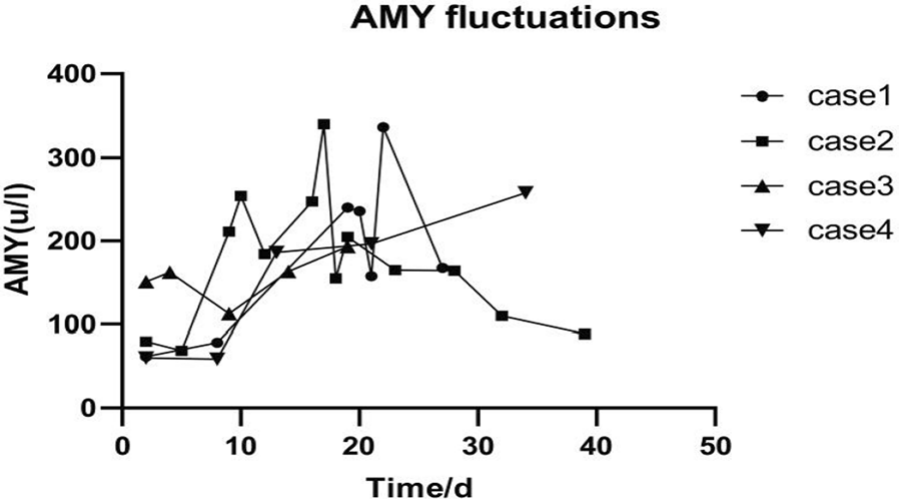
AMY fluctuations of the four cases and the corresponding course of disease. The four cases started to use high-dose mPSL on the 3rd day, 6th day, 8th day, and 9th day of the disease course. AMY elevation was noted on the 19th day, 9th day, 14th day, and 13th day, respectively. AMY peak appeared on day 22, day 17, day 18, and day 34, respectively. Amylase was monitored for five consecutive days in cases 1 and 2 and weekly in cases 3 and 4

After high-dose intravenous administration of mPSL, serum amylase (AMY) levels increased in four patients, peaking at 193.1 to 340.4 U/L. In addition, increases in urine amylase and serum lipase were also observed. Abdominal enhanced CT scan performed after the occurrence of hyperamylasemia in case 1 showed sand-like stones in the gallbladder, with a normal shape of pancreas. In cases 2, 3, and 4, abdominal ultrasound performed prior to high-dose mPSL, indicated gallbladder sediment-like stones, rough gallbladder wall, and gallbladder stones, respectively. Notably, the CT scans in cases 2, 3, and 4 did not reveal any abnormalities of the pancreas. The AMY fluctuations of the four patients over time are shown in Fig. 1.

#### Diagnostic assessment and diagnosis

The above clinical manifestations, physical examinations, and clinical data were to be not very specific for diagnosis. Only cases 1 and 2 experienced epigastric pain, while enhancement CT in case 1 revealed gallstones. We were able to diagnose hyperamylasemia on the basis of the fact that the four patients had different elevations of AMY that exceeded the normal reference values of our hospital system. We speculate about the possibility of hyperamylasemia caused by high doses of intravenous GC through the adverse drug response evaluation described below. We considered whether this can be attributed to drug-induced pancreatitis, but imaging did not reveal any pancreatic abnormalities and therefore diagnostic criteria for pancreatitis were not met. The overall prognosis of the four patients is good.

### Interventions and outcomes

Case 1 and 2 underwent fasting. Then, they were given omeprazole to inhibit gastric acid, phloroglucinol as antispasmodic, somatostatin to inhibit pancreatic enzyme secretion, and anti-infection and nutritional support treatment. Two patients were discharged after a significant decrease in AMY levels, and case 3 and 4 were discharged after improvement of the primary disease. At outpatient review, they indicated that mPLS was effective for the primary disease and that, despite the unexpected increase in AMY, subsequent active interventions led to normalization of AMY. Unfortunately, they have not been followed since then.

## Discussion

Hyperamylasemia can be divided into four types: pancreatic hyperamylasemia, salivary gland hyperamylasemia, megaamylasemia, and mixed hyperamylasemia [7], being mainly due to pathological changes in the organs that secrete AMY and impaired metabolic clearance. Common causes such as acute pancreatitis, pancreatic or salivary gland trauma, ERCP, etc., lead to gland storage AMY secretion to the blood, resulting in hyperamylasemia. AMY is cleared through the kidney and reticuloendothelial system in the human body, and hyperstarchemia occurs in renal insufficiency and liver disease (hepatitis or cirrhosis) [5]. Studies have shown that AMY is increased in patients with chronic renal insufficiency when eGFR < 60 ml/min/1.73 m^2^, and AMY increased level was negatively correlated with eGFR [8]. Cases 1 and 2 had normal renal function throughout the disease, while cases 3 and 4 had renal insufficiency, especially case 4, which could have been diagnosed as uremia, relying on hemodialysis for a long period of time to maintain renal function. On outpatient follow-up, we observed a normalization of amylase in the latter two, but there has been no significant recovery of renal function. Therefore, the possibility of increased amylase due to renal insufficiency was temporarily ruled out. The renal function results of the four cases are presented in Table 2.

**Table 2:** Renal function results of the four cases

Day	AMY (U/L)	CREA (umol/L)	UA (umol/L)	eGFR (mL/min/1.73 m^2^)
Case 1				
2	61.9	45.5	142.1	113
8	78.4	40.2	164	117.7
18	233.6	39	120	118.9
27	167.6	36.2	142.7	121.8
Case 2				
2	79.9	61.3	153	120.4
5	68.7	44.8	125.7	133.5
9	211.2	46.1	107.9	132.2
32	109.9	39.7	258.6	138.9
Case3				
1	150.8	98.1	486.4	57.8
4	162.6	85.9	456.5	67.9
9	112.5	129.5	571	41.3
14	163	105.4	474.1	53
18	193.1	105.2	372.8	53.1
Case 4				
1	60.1	466.3	297.2	7.6
8	58.7	260.2	206.1	15.4
13	186	343.6	272	11
21	196.4	385.6	319.6	9.8
34	257.9	345.4	404.1	11

*CREA creatinine, *UA* uric acid, *eGFR* estimated glomerular filtration rate

Moreover, previous studies have reported that systemic lupus erythematosus is associated with elevated AMY, which can be attributed to the production of autoantibodies, immune process activation, and vasculitis-induced lupus pancreatitis [9]. Case 3, in which amylase was above normal on admission, was considered to be related to SLE. The amylase then decreased to normal with low doses of mPSL and cyclophosphamide. SLE was effectively treated with high-dose mPSL from day 8, after which amylase increased again, which was considered to be related to the high-dose mPSL. Interestingly, when case 3 was admitted for the second time, 60 mg of mPSL was administered for 13 consecutive days and a slight increase in amylase was noted.

Gallstone is the common cause of acute pancreatitis. In these four cases, we could almost observe gallbladder sediment-like stones, but before the application of high-dose glucocorticoid, amylase was normal, and there were no relevant imaging manifestations of acute pancreatitis, so we can reject the influence of gallbladder stones.

In this study, the relationship between mPSL and hyperamylasemia was evaluated by the Naranjo scoring method [10], as shown in Table 3, and the following is a detailed description of the contents of the rating scale. (1) According to literature, Logsdon *et al.* reported that GC induced synthesis of AMY by AR42J pancreatic acinar cells in 1985 [11], indicating that GC could cause an increase of amylase. (2) In addition, except for case 3, the other cases had normal AMY levels at admission, and hyperamylasemia occurred only after high dose of mPSL. (3) In cases 1 and 2, AMY secretion was inhibited by somatostatin, and hyperamylasemia was relieved. (4) The fluctuation of amylase can be used as objective evidence, and other possible causes of hyperamylasemia are excluded in the discussion above, indicating that effective diagnosis of hyperamylasemia was performed.

**Table 3:** Naranjo scale questionnaire

	Naranjo questions	Yes	No	Do not know
a	Are there previous conclusive reports on this reaction?	1	0	0
b	Did the adverse event occur after the suspected drug was administered?	2	−1	0
c	Did the adverse reaction improve when the drug was discontinued or a specific antagonist was administered?	1	0	0
d	Did the adverse reaction reappear when the drug was readministered?	2	−1	0
e	Are there alternative causes (other than the drug) that could have on their own cause the reaction?	−1	2	0
f	Did the reaction reappear when a placebo was given?	−1	1	0
g	Was the drug detected in the blood (or other fluids) in concentrations known to be toxic?	1	0	0
h	Was the reaction more severe when the dose was increased or less severe when the dose was decreased?	1	0	0
i	Did the patient have a similar reaction to the same or similar drugs in any previous exposure?	1	0	0
j	Was the adverse event confirmed by any objective evidence?	1	0	0

However, our research also has some limitations. For example, there was no mention of similar adverse drug reactions in patients’ previous medical history nor high dose of GC rechallenge. In this study, we did not carry out placebo-controlled trials or monitor blood concentrations after addition and subtraction doses to compare the severity of hyperamylasemia. Due to the failure of follow-up, we only know that the AMY of the four patients returned to normal after the first outpatient review, so it is impossible to predict whether hyperamylasemia will recur.

In summary, the total score for the above evaluation criteria was 7 points, indicating that the causal relationship of hyperamylasemia caused by high-dose mPSL is “very likely.”

Previous studies have reported that GCs can lead to acute pancreatitis [12–14]. China’s latest guidelines [15] suggest that the diagnosis of acute pancreatitis should meet at least two of the following three criteria: (1) acute, sudden, persistent, severe upper abdominal pain, which may radiate to the back; (2) serum amylase and/or lipase activities at least three times higher than the upper limit of normal levels; (3) enhanced CT/MRI showing typical AP imaging changes (pancreatic edema or peripancreatic effusion). These four cases did not meet the diagnostic criteria for pancreatitis, so the diagnosis was a simple increase in AMY caused by drugs, which might be a direct toxicity of mPSL.

## Conclusion

Elevated amylase levels may be a result of pancreatitis or a reflection of nonpancreatic disease. Simple hyperamylasemia is clinically insignificant and does not require treatment. However, etiological differentiation of hyperamylasemia should be emphasized in clinical practice, especially when the diagnosis of acute pancreatitis is not clear. The hyperamylasemia in this study was most likely caused by acute pancreatitis, as the diagnosis has not yet been reached. It is recommended to increase tests of serum lipase, giant amylase, and pancreatic amylase isoenzyme as well as imaging examinations of pancreas when necessary, and to follow up outpatients with high amylase.

## Data Availability

All data generated or used during the study appear in the submitted article.
